# Speeding genomic island discovery through systematic design of reference database composition

**DOI:** 10.1371/journal.pone.0298641

**Published:** 2024-03-13

**Authors:** Steven L. Yu, Catherine M. Mageeney, Fatema Shormin, Noushin Ghaffari, Kelly P. Williams

**Affiliations:** 1 Sandia National Labs, Livermore, California, United States of America; 2 Department of Computer Science, Roy G. Perry College of Engineering, Prairie View A&M University, Prairie View, Texas, United States of America; Wilfrid Laurier University, CANADA

## Abstract

**Background:**

Genomic islands (GIs) are mobile genetic elements that integrate site-specifically into bacterial chromosomes, bearing genes that affect phenotypes such as pathogenicity and metabolism. GIs typically occur sporadically among related bacterial strains, enabling comparative genomic approaches to GI identification. For a candidate GI in a query genome, the number of reference genomes with a precise deletion of the GI serves as a support value for the GI. Our comparative software for GI identification was slowed by our original use of large reference genome databases (DBs). Here we explore smaller species-focused DBs.

**Results:**

With increasing DB size, recovery of our reliable prophage GI calls reached a plateau, while recovery of less reliable GI calls (FPs) increased rapidly as DB sizes exceeded ~500 genomes; i.e., overlarge DBs can increase FP rates. Paradoxically, relative to prophages, FPs were both more frequently supported only by genomes outside the species and more frequently supported only by genomes inside the species; this may be due to their generally lower support values. Setting a DB size limit for our **SMA**ll **R**anked **T**ailored (SMART) DB design speeded runtime ~65-fold. Strictly intra-species DBs would tend to lower yields of prophages for small species (with few genomes available); simulations with large species showed that this could be partially overcome by reaching outside the species to closely related taxa, without an FP burden. Employing such taxonomic outreach in DB design generated redundancy in the DB set; as few as 2984 DBs were needed to cover all 47894 prokaryotic species.

**Conclusions:**

Runtime decreased dramatically with SMART DB design, with only minor losses of prophages. We also describe potential utility in other comparative genomics projects.

## Introduction

The comparative approach is one of the oldest and most powerful methods in biology, expressed thus by Aristotle: “first we must grasp the differences, then try to discover the causes” (*History of Animals* I.6). For any given trait under study, there is an appropriate degree of relatedness among the compared organisms, that will reveal the similarities and differences required to best define the trait. Comparative genomics, being typically computational, is often formulated as a comparison of a query genome to one or more reference genomes, i.e., a reference genome database (DB). We have used comparative genomics [1, 2] for mapping genomic islands (GIs); these are mobile genetic elements that play critical roles in evolution, integrating at specific sites in bacterial and archaeal chromosomes and bearing genes affecting traits such as pathogenicity and metabolism. We find that the taxonomic level of species is often appropriate for comparative GI mapping; for any GI in a query genome, another genome from the species can often be found that lacks the GI, providing the Aristotelian difference required to identify and precisely map the GI. However, some species have few genomes available, and in these cases reaching to higher taxa may be required to apply comparative genomics. The systematically reconstituted bacterial and archaeal taxonomic system of the Genome Taxonomy DataBase (GTDB) project [3] aids such work.

Our comparative genomic software, TIGER [1], identifies and precisely maps GIs through a ping-pong application of BLASTN [4]: segments of the query genome likely to contain one end of a candidate GI are used to search a reference genomic DB, and hits are used to collect reference sequences that are then searched back to the query genome to find the other end of the GI. Reference genomes that map a GI call presumably have an intact (uninterrupted by any GI) integration site for the GI; the number of such reference genomes serves as a support metric for the GI. This approach misses ancient GIs that may have lost key features (their integrase gene or their flanking attachment sites), but the numerous GIs that it does find are more likely to be actively mobile. Despite the success of TIGER in precise mapping of numerous GIs in bacterial and archaeal genomes, false positives can also arise, perhaps from other chromosomal rearrangement events that occurred in a small number of reference genomes. Execution of TIGER was often slow and we suspected this was due to our original choice to use a small number of very large reference DBs (Methods). Here we explore alternatives involving small species-focused DBs, and the consequences for GI yields.

Different DB design problems arise for large (many genomes available) and small species. For large species, should we draw DB genomes only from within the species and is there an optimal number of genomes? For small species, should we draw additional genomes from outside the species, and if so, to what phylogenetic/taxonomic extent? An algorithm was developed for constructing a DB set that covers all species of any GTDB release. Because it allows spread to higher taxa for smaller species, it produces redundancy in the DB set; as few as 2984 DBs were needed to cover all 47894 species of GTDB release 202. These new species-tailored DBs are far smaller than those we used previously, allowing our comparative software to run much faster. Aside from mobile element detection, these DBs can serve other comparative genomic goals, such as within-species survey of any genomic region of interest. We present software for revision of the DB set upon GTDB update.

## Results

### Smaller DBs for large species

With our original large reference DBs (Table 1), TIGER performance in identifying GIs was slow, motivating an exploration of alternative DB composition. For the 195,890 properly treated genomes (Methods), we estimate based on the benchmarking studies presented below that a total of 18.0 CPU years was spent on the BLASTN and subsequent TIGER steps. On the other hand the large DBs provided much information on how often distantly related genomes support a GI. Because different GI types may respond differently to DB composition, we sorted each GI call based on gene content into various types, such as Phage, Integrative Conjugative Element (ICE), or Non-Phage/Non-ICE (NonPI) [1]. The ICE type was further split into ICE1 (more ICE-like) and ICE2 (less ICE-like). Similarly, the Phage type was split into Phage1 and Phage2, as well a third category PhageFil (filamentous). Here we introduce a new type “Reject” for any GI call with size or other characteristics (specified in Methods) that our spot checks have never found to produce convincing GIs; we consider these to be nearly all false positives. Although Reject calls could easily be immediately discarded, study of their properties may help uncover additional false positives. They also serve as negative controls for understanding some of the results presented below. In contrast, we consider the Phage1 GIs (prophages) to be nearly all true positives based on previous benchmarking against gold standards [1] and experimental results on inducibility [5]. Support values (number of genomes found with precise deletion of the GI) are substantially different for these two types, averaging 5212 for the prophages, but only 700 for the Rejects. The largest category of GIs, NonPI, has an intermediate average support value of 1999.

**Table 1:** Original large DBs. DBs were assembled based on taxonomy as assigned at NCBI, aiming for roughly even distribution of all genomes collected. After assignment to GTDB r202 species, small numbers of genomes were found to be improperly treated by placement in a different large DB than the bulk of the species’ genomes; these improperly placed genomes were excluded from further analysis and are not counted in the Species or Genomes columns. The two DBs limited to genus (Salmonella and Campylobacter) were not treated further because taxonomic outreach could not be studied, leaving 11 DBs studied herein, with totals at bottom. For these 11 DBs, the genomes treated and species composition are reported in S1 and S2 Files in S1 Data, respectively.

Large DB	Species	Genomes	Rank Limit	Genera with Large Species
Salmonella	5	58701	Genus	
EnterobacteriaceaeOther	210	27850	Family	*Escherichia*, *Klebsiella*, *Enterobacter*, *Cronobacter*
EnterobacteralesOther	343	2469	Order	
GammaproteobacteriaOther	3255	24631	Class	*Vibrio*, *Pseudomonas*, *Acinetobacter*
Campylobacter	89	30629	Genus	
EpsilonproteobacteriaOther	221	1927	Class	
ProteobacteriaOther	5611	18799	Phylum	*Burkholderia*
Actinobacteria	3798	17613	Phylum	*Mycobacterium*
Listeriaceae	25	21995	Family	*Listeria*
Streptococcaceae	290	27907	Family	*Streptococcus*
FirmicutesOther	4848	32779	Phylum	*Bacillus*, *Enterococcus*, *Clostridioides*, *Staphylococcus*, *Lactiplantibacillus*
BacteriaOther	7963	17258	Division	
Archaea	1435	2662	Division	
Total, excluding Salm./Camp.	27999	195890		

We began by examining a species with numerous genomes available, *Escherichia flexneri* (herein we employ species names from GTDB release 202; this is the species containing the classical *E*. *coli* K-12 strains). We collected 9089 genome assemblies explicitly included in *E*. *flexneri* by GTDB and 843 additional genomes placed in the species by our assignment software (Methods). TIGER was applied to all these query genomes using our original large DB containing 27868 genomes from the Enterobacteriaceae family (Table 1). TIGER output provides for each GI call a list of all the reference genomes that supported the GI. For each GI we could use its list of supporting genomes to measure the power of various smaller (subset) DBs to recover the GI. To test effects of reference DB size, an ordered list of the GTDB genomes was prepared either by random shuffling or by a ranking algorithm partly involving genome assembly quality and mainly based on genome diversity (Methods). Then nested sets of various sizes from the top of the list were collected and evaluated for GI recovery.

**Fig 1:**
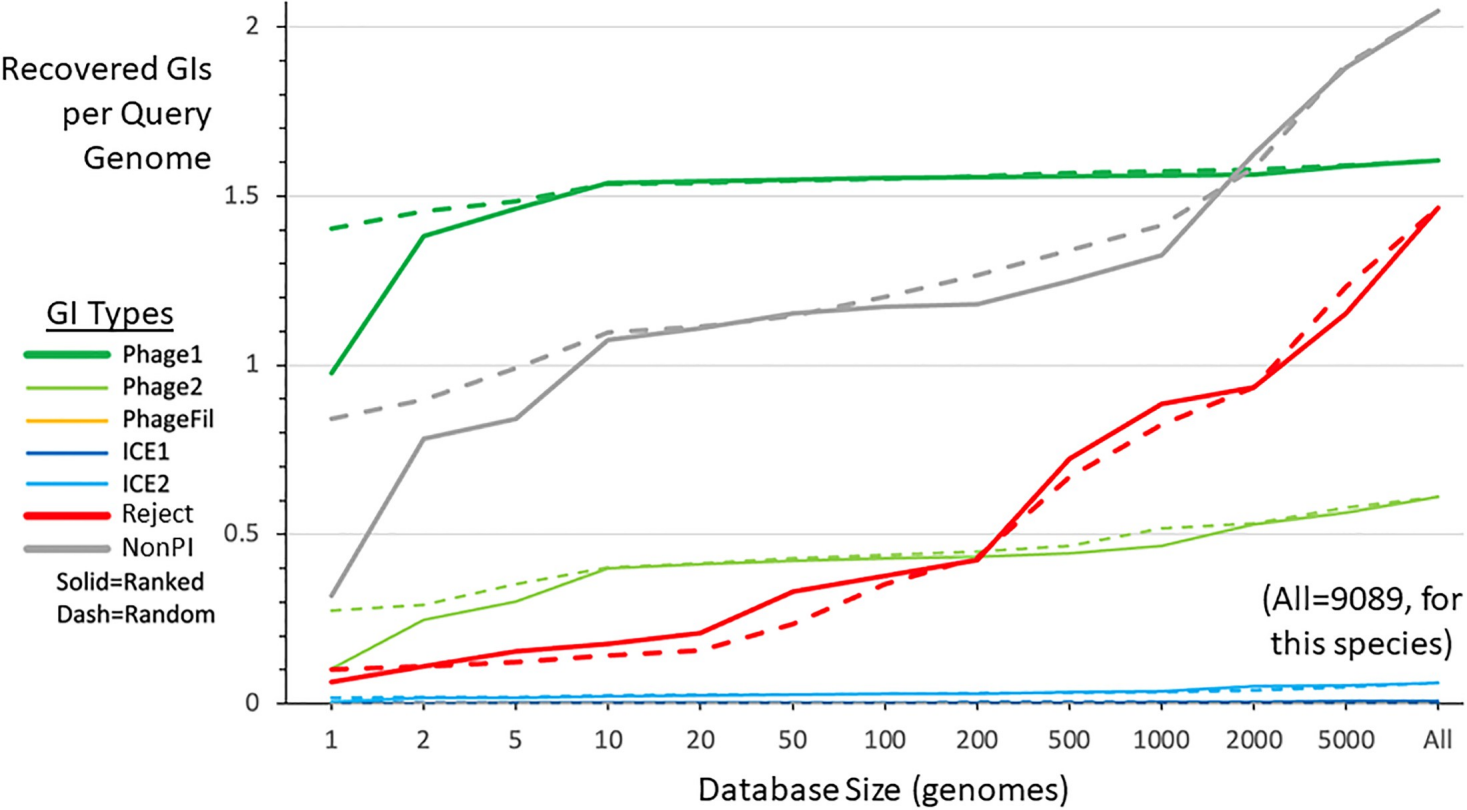
*E*. *flexneri* GI calls recovered with various DB sizes. The GI identification program TIGER was run on 9932 *E*. *flexneri* query genomes using the large reference DB Enterobacteriaceae that contained a set (“All”) of 9089 reference *E*. *flexneri* genomes. GI calls were typed, and those calls that either had no support from All or were in tandem arrays were discarded. DBs of various sizes that were subsets of All, were designed using the random or ranked protocols (Methods). Average count of GIs recovered per query genome (supported by at least one genome in the test DB) were taken for each GI type. Here, the PhageFil lines are obscured by the ICE1 lines.

Fig 1 explores the use of subsets from the 9089 GTDB *E*. *flexneri* genomes to recover each GI, reporting the average GI count per query genome for each GI type, as database size is varied. Recovery of prophages (a large group of particularly reliable calls, marked Phage1) plateaus, with few added as DB size rises above 200. In contrast the Reject category of unreliable calls rises continuously, especially with DB size above 200. The similar behavior of random and ranked composition methods shows that rises at high DB size are not due to the concentration of lower quality genomes in the largest ranked DB.

Like the Rejects, the NonPI category shows a rise in recovery above DB size 200, suggesting that this category contains some false positives. However, NonPIs are intermediate between Phage1s and Rejects by multiple metrics, suggesting that they also include legitimate GIs. To examine these hypotheses, we manually inspected 70 large NonPI families (based on integration site usage) from seven diverse large species. Inspection suggested that most were valid, with predicted genes for metabolism cassettes, restriction enzymes, and toxin/anti-toxin addiction cassettes, among others. However, 10 of the GI families examined (14.3%) were apparent false positives, distinguished by such gene content features as numerous transposase genes, numerous housekeeping genes, or only a portion of a single gene (the integrase gene itself).

**Fig 2:**
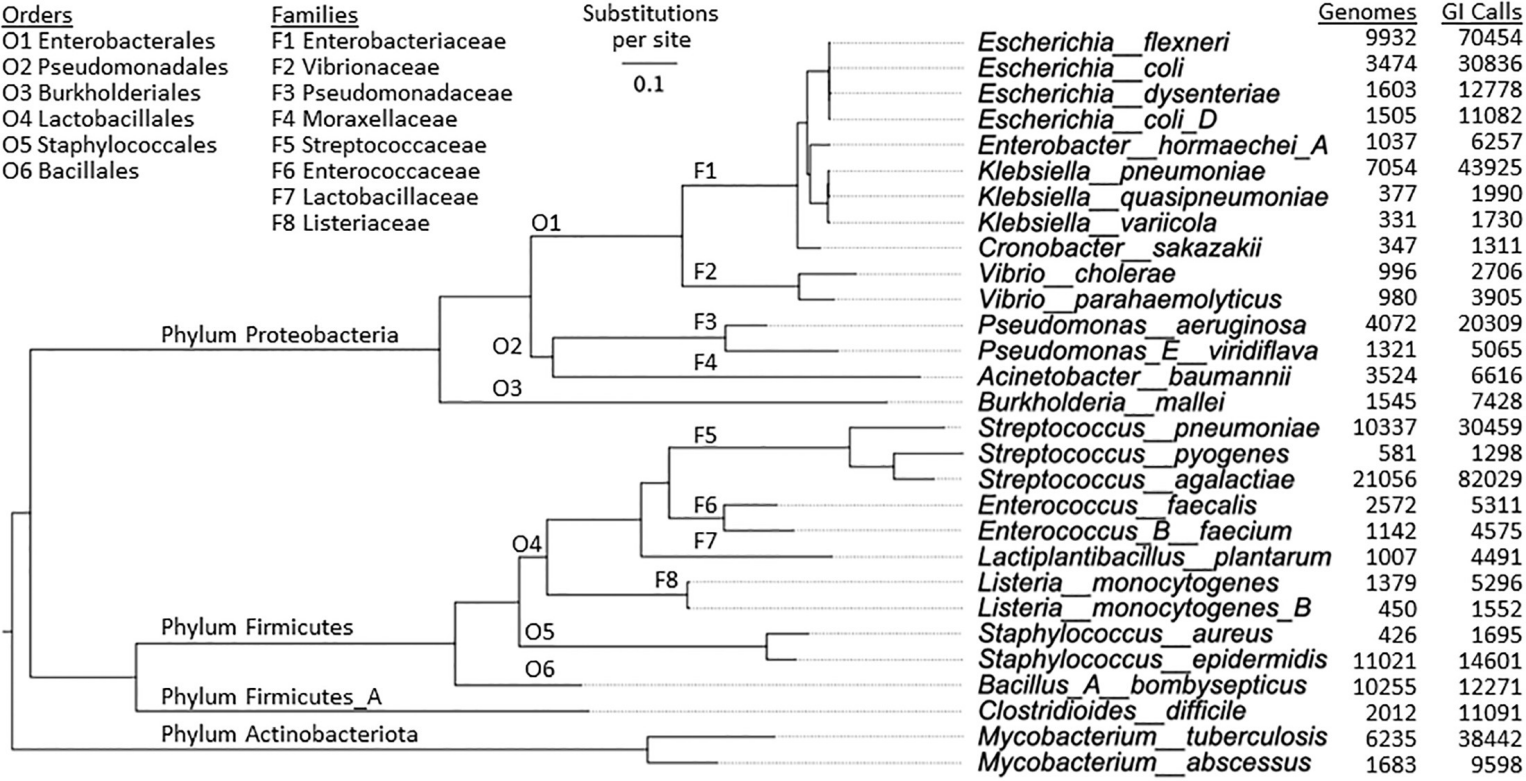
Large species studied herein. The bacterial species representative tree from GTDB release 202 was pared to the 29 large species of interest using our wrapper (pare_tree_gtdb) for PareTree (http://emmahodcroft.com/PareTree.html) and visualized in FigTree (http://tree.bio.ed.ac.uk/software/figtree). GTDB has subsumed the standard class for *Burkholderia* (Betaproteobacteria) into the Gammaproteobacteria.

To examine these trends in other large species, we identified all 29 species with sufficient yields of the key GI types: prophage, Reject and NonPI (Methods and S1 Table in S1 Data); these were pathogen species from the phyla Proteobacteria, Firmicutes and Actinobacteria (Fig 2). For each species, sets of smaller DBs were prepared and islands analyzed as for *E*. *flexneri* (S1A-S1H Fig in S1 Data). Similar trends to those for *E*. *flexneri* were observed for the other large species, although for species with fewer than 500 genomes, the large-DB rise in Reject and NonPI GIs is muted. Same-genus species pairs sometimes differ in absolute yields and curve shapes of the key GI types, especially for the NonPI category (e.g., *E*. *coli* vs. *E*. *flexneri*), perhaps due to high strain specificity and species penetration of individual GIs.

For the large species combined, limiting DB composition to genomes within the species had a small cost, resulting in the loss of 1.4% (1384 total) of all their prophages (S2 Table in S1 Data, line 30). However these lost prophage GIs generally had low support values, usually of 5 or less. It would thus be difficult to use simple principles to design small DBs that could support these lost prophages. Moreover, limiting composition to within-species had a positive effect, eliminating a far higher fraction (20%) of the Reject GIs. NonPI GI loss is intermediate; thus losses from within-species DB composition may simply correlate with average support values. This shows that limiting DB composition to members of the same species is valuable, at least when genome numbers are sufficiently high.

### Expanding DBs to higher taxa for small species

Most species have insufficient genomes to fill DBs of size 200. Indeed, GTDB lists only a single genome for 30777 of 47894 species. An option for small species is to fill their DBs by reaching beyond the species to higher taxa. This raises questions of whether such outreach would be effective, whether there is a reasonable taxonomic limit for such reach, and whether false positive rates might become unacceptable. The above result, that the Rejects had a much larger fraction than did the prophages of GIs with support only from outside the species, might indicate that false positives would be favored by reaching to higher taxa. To investigate these questions, we used the data-rich large species to simulate small species, by omitting all support from genomes within the same species. Surprisingly this showed that Reject GIs more frequently had support only from inside the species (S2 Table in S1 Data, line 30), over two-fold more often than for prophages. The seemingly opposing results, that Rejects were more frequently supported only from outside the species and were more frequently supported only from inside the species, may both be due to the low average support values of Reject GIs. These trends generally (but not always) hold for individual large species.

**Fig 3:**
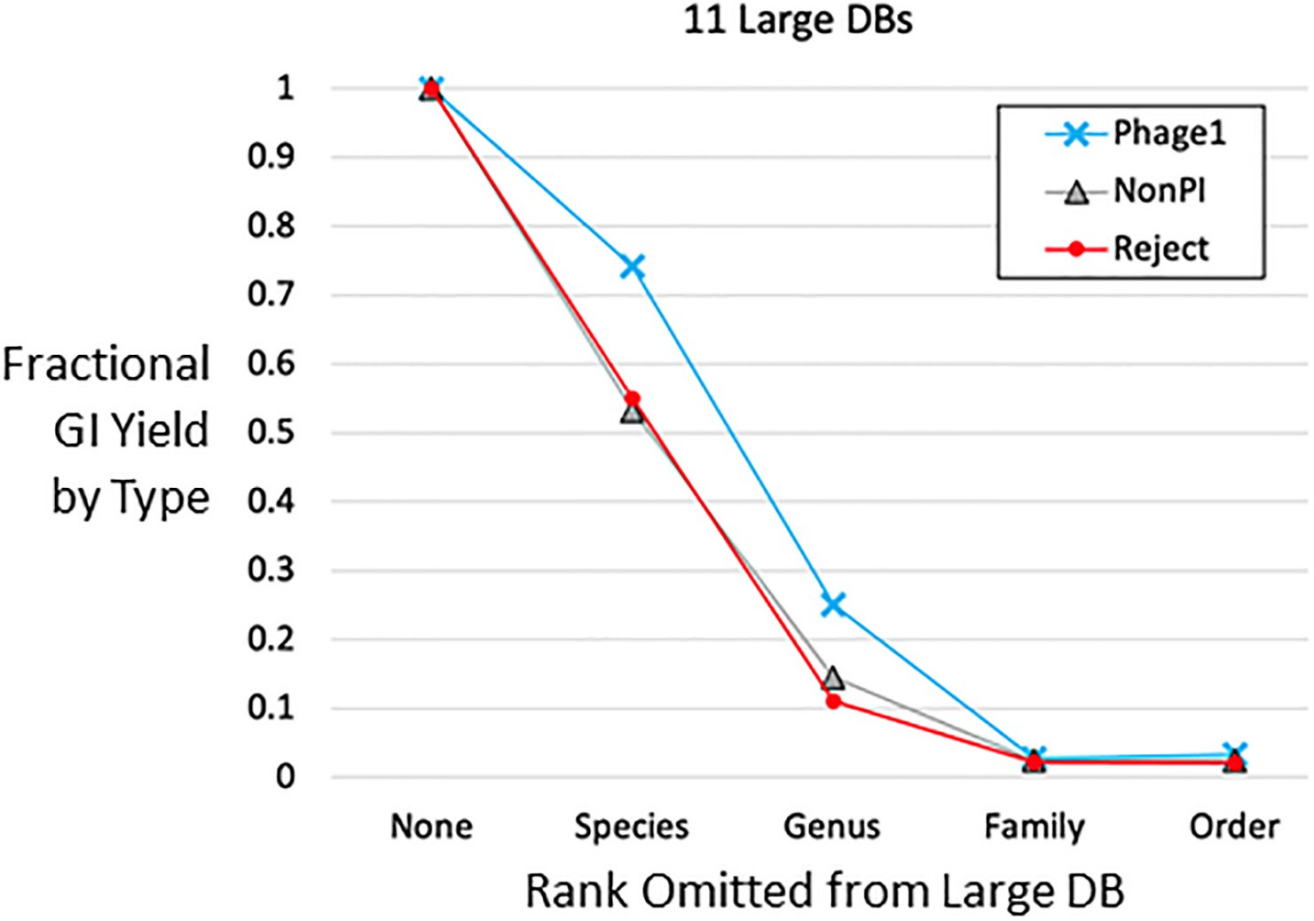
Utility of higher taxa for GI detection. For each GI in the full set, the list of supporting genomes from the large DB was filtered by removing all genomes from the same species, or from the same genus, family or order. This simulates small species that may have no other genomes available from the same species, or the same genus, etc. For the three large DBs that are limited to a single family, all GIs are lost when omitting same-family genomes, so the GIs from these DBs were excluded from the denominator for the family-omission and order-omission treatments. GIs from the single-order DB EnterobacteralesOther were similarly excluded from the denominator of the order-omission treatment.

For each large species, GI yields were substantial after excluding support from the same species itself and, as noted for all large species combined, losses for the desirable Phage1 GIs were low while losses for the undesirable Reject GIs were high. These trends showed promise for use of out-species reference genomes for small species. We tested further taxonomic reach by omitting support from higher taxa: omitting all support from within the genus, or from within the family, or from within the order. Fig 3 shows the combined result for the entire GI set. Yields drop to 2–3% for family and order omissions, but Phage1 fractional yields are higher than for Rejects at each omission step.

**Fig 4:**
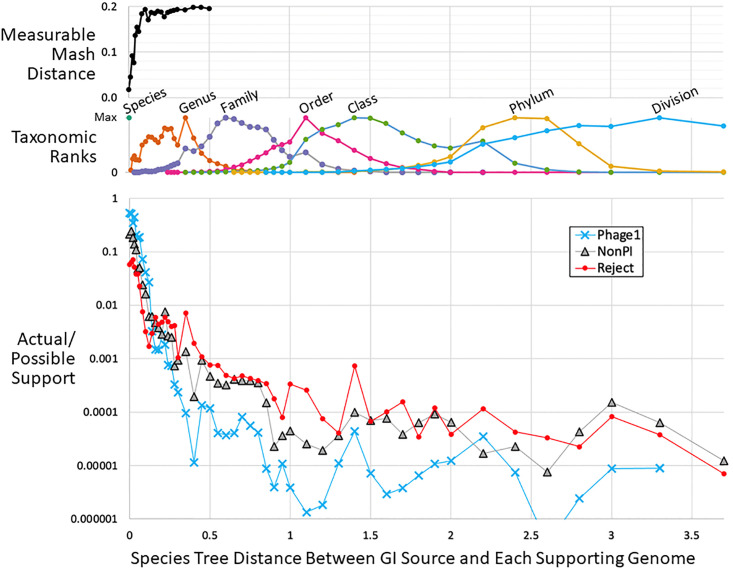
Actual/possible support. Main panel C: For each GI (separately treating the three main types: Phage1, NonPI and Reject), there was list of possible supporting genomes, i.e. all the genomes in the large DB used to evaluate it. The tree distance (substitutions per site) was taken from the GI’s source genome to all possible supporting genomes based on the multi-protein species trees of GTDB release 202; intra-species genome pairs always receive a distance score of zero. The possible support distance counts were placed into 50 bins from 0 to 3.7. TIGER also reports a list of actual supporting genomes for each GI, whose distances to the GI source genome were likewise taken. To aggregate data for all islands, the actual and possible support counts were summed (by type) in each bin. Finally actual support totals were divided by possible support totals for each bin. Note the logarithmic y-axis. Panel B: For each genome pair in every large DB, the shared taxonomic rank was taken, and species distances tallied (middle panel, with counts for each rank’s trace normalized to the maximum count in the trace). Panel A: For each genome pair, Mash distances at or below the reliability threshold (0.2) were taken and binned by species distance; by tree distance 0.2, Mash distance has plateaued at its maximum, after which percentages of measurable genome pairs decrease until cutting off reporting after tree distance 0.5.

Fig 3 was based on taxonomic ranks, but we were also able to examine GI support decay with the finer criterion of phylogenetic distance, using the bacterial and archaeal species trees provided by GTDB; these trees are robust, based on ~100 proteins for each species representative. Fig 4 shows actual/possible support values plotted against phylogenetic distance (panel C), comparing with the shared taxonomic rank and Mash distance. Mash distance (panel A) is a useful metric within a species or its close relatives, whereas the phylogenetic species distance (panel B) is zero and therefore uninformative for same-species genomes but becomes a useful metric when comparing genomes from different species or higher taxa. In panel C, the y-axis (species distance) value of zero corresponds to using only references genomes from the same species as the query genome, allowing us to assess the effect of a possible DB-building rule where only same-species genomes are allowed. Thus mimicking same-species-only DBs, we observe that actual/possible support is lowest for the Reject type and highest for Phage1. There is a large drop in actual/possible support as we move from using same-species genomes to using genomes from other species in the same genus. At approximately the genus level of distance (phylogenetic distance ~0.4), the Reject/Phage1 trend reverses and Reject GIs receive more actual/possible support than Phage1. This result differs from the Fig 3 result on fractional yields, and reveals a cost to using more distant reference genomes, although this may be acceptable when very few closely related genomes are available.

### SMART DB software

**Fig 5:**
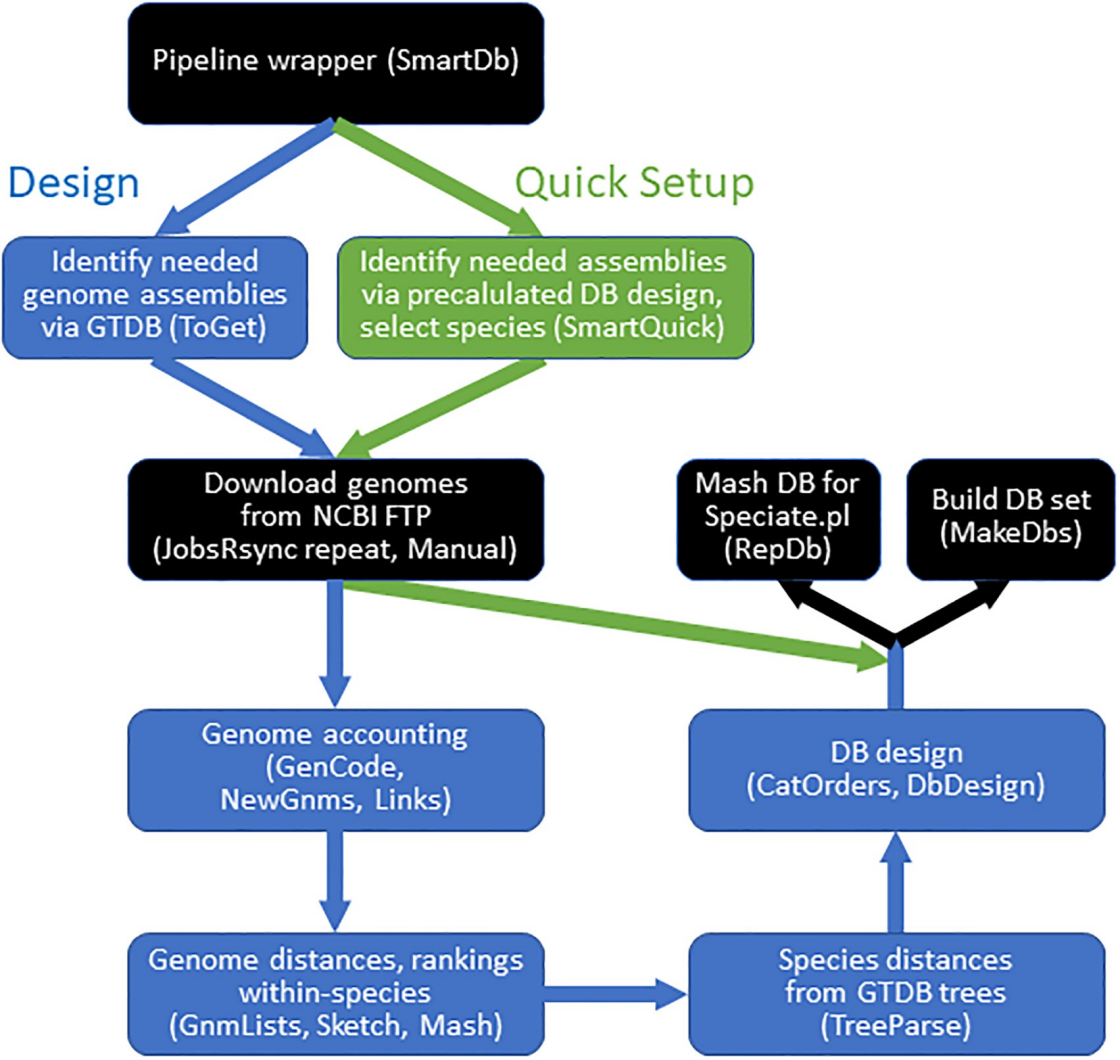
SMART DB software pipeline. As described in Methods, the Design mode (blue) operates on an initial GTDB release or update, collects needed genomes, and designs and builds the DB set. The Quick Update mode starts with a precalculated DB design file (and an optional list of desired species) and builds DBs. Scripts employed at each step (and a potential manual genome collection phase) are in parentheses.

Based on the above considerations we settled on the following design scheme for **SMA**ll **R**anked **T**ailored (SMART) DBs: 1) DB size is capped at a maximum number of genomes; here we tested maxima of 200, 300 and 500, based on the performance in this range observed in Fig 1 and S1. 2) This cap is reached phylogenetically, meaning that after exhausting the same-species ranked genome list, genomes from additional species are brought in according to species distance taken from the GTDB tree. 3) Phylogenetic filling is limited to the taxonomic rank of order, which corresponds to what remains after omitting same-family genomes (the category “Family” in Fig 3). We have developed the SMARTDB software pipeline (Fig 5) that automates design and preparation of DBs for all species, or as many species as desired, including the step of collecting any needed genome assemblies. Benchmarking studies for three genomes from each large species (S2 Fig in S1 Data) showed useful speedup from capping DB size; the within-species DBs capped at 200 genomes had an average speedup of 2.4-fold relative to those capped at 500 genomes, and 65-fold relative to the original large DBs.

Our original set of GTDB-assigned genomes covered only 28500 of the 47894 species. We ran our software to collect additional genomes, increasing our collection to 348,547 that included all genomes known for GTDB release 202 species with fewer than 500 genomes, and at least 500 genomes each for the larger species. Designing the SMART DB set for all GTDB species, we observed redundancy; the design feature of phylogenetic reach produced many mixed-species DBs, and moreover many cases where the same mixed-species DB serves multiple species. As few as 2984 unique DBs were needed to cover all 47894 prokaryotic species (Table 2, line 3). Because of the cap on DB size, only 55.7% of available genomes were needed to build all DBs.

**Table 2:** SMART DB sets for two GTDB releases. Submaximal DBs were those containing fewer genomes than the maximum. Taxonomic filling was stopped at the rank of order.

GTDB release	Species	DB size maximum	Unique DBs	Submaximal DBs	DB size < 5	Unique genomes
202	47894	200	4345	1234	598	144858
202	47894	300	3633	1247	598	151618
202	47894	500	2984	1267	598	160724
207	65703	200	5563	1489	735	188952
207	65703	300	4627	1513	735	196780
207	65703	500	3721	1538	735	206636
214	85205	200	7042	1680	798	244378
214	85205	300	5789	1710	798	254878
214	85205	500	4619	1736	798	267042

The GTDB project is ongoing and produces occasional updates that tend to change taxonomic assignments and increase species numbers. After the work herein was initiated, GTDB updated to releases 207 and 214, increasing the species count. The SMART DB pipeline operates in an update mode, and we used this to design SMART DB sets for the new releases (Table 2), with similar conclusions as for the earlier release. In addition to the *de novo* and update Design modes, our pipeline operates in a Quick Update mode, where the user can begin with our precalculated DB design file to avoid GTDB file download and the design calculation phase.

## Discussion

The problem of identifying GIs through the comparative approach is important in its own right, but also stands in for similar problems about other classes of mobile genetic elements and, more broadly, about the “accessory” (non-core) fraction of the pan-genome for a prokaryotic species. In principle, a single genome per species could suffice for GI finding; the GTDB system already designates a single representative genome for each species. At another extreme, a large reference DB containing all available genomes for a species, and perhaps beyond the species, could be used. Here, to our knowledge for the first time, we systematically evaluate these simple DB design strategies and intermediate alternatives in terms of recovery, false discovery rate, and efficiency. We show that use of a single reference genome generally suffers from low recovery, while overlarge DBs can yield excess false positives and slow runtimes. When numerous genomes are available for a species, DB sizes of ~200 diverse genomes provide an excellent balance between recovery of true positives, avoidance of false positives, and fast runtimes. An algorithm, supported by software, is presented for preparing a high-performance reference genome DB for any prokaryotic species.

Our category “Reject”, nearly all false positives, is readily defined and can quickly be filtered out. However, retaining them was useful for this study, revealing characteristics that should help identify and remove additional false positives among other groups, especially the NonPIs. We observed that Reject GIs were more frequently supported only by genomes outside the species (20.5%) than were the reliable Phage1 GIs (1.4%). Paradoxically, Reject PIs were also more frequently supported only by genomes *inside* the species (49.0%) than were Phage1 GIs (20.5%). Rearrangement of a reference genome chromosome by mechanisms other than integrase action can lead to false positive GI calls, and such rare rearrangements may occur either within the species or in reasonably close relatives outside the species. This rare false positive explanation is borne out by statistics; average support values of Rejects are simply much lower (7.45-fold) than those of the prophages.

In some ways the work herein is particular to GIs and to our software for their detection. For example, TIGER demands BLASTN hits of > = 500 bp at both flanks of each GI; more taxonomically distant reference genomes tend to fall below BLASTN detection limits. Nonetheless, with the simple design principles of the SMART DB sets, we envision additional uses in other comparative genomics applications. Our TIGER software is also capable of mapping the set of transposable elements (TEs) within a genome [1]. Faster performance now enables survey of TEs among prokaryotes. There are 2.4-fold more transposases than integrases among our genomes; applying the formulae of S2 Fig in S1 Data to each of the properly treated genomes, we estimate that transposable element search completion would take 26.5 CPU years with the old large DBs, but only 0.78 CPU years with the 200-genome SMART DB set. Further speedup may be possible using alternatives to BLASTN or improved hardware architecture [6]. The DBs may also help survey such highly variable gene sets as the capsule and lipopolysaccharide clusters of Proteobacteria [7]. Precise mapping of the boundaries of such gene clusters may help reveal mechanisms of their variability, even when recombination enzymes such as integrases do not appear to explain their evolution. More broadly the DBs should serve the endeavor to define the two main genomic fractions of a species pan-genome [8, 9], the conserved core and the sporadically-occurring accessory genome that includes GIs. The SMART DBs can be said to well define the core genome of the species. We found that ~200 genomes from a species are generally sufficient to provide the integration site of each GI in its uninterrupted form. Certainly there are species-defining genomic islands that might be missed by our species-limited DBs, such as the SPI1 and SPI2 of *Salmonella enterica*, but these are not found by TIGER anyway; they are so ancient that they have lost their mobility function, including the integration module, and moreover have lost the sharp borders sought by TIGER [10].

## Conclusion

We show that species-focused reference DBs capped at 200 genomes are sufficient to recover most high quality GIs while precluding some low quality GIs. They greatly speed GI search software. For species with fewer genomes available, effective DBs can be built by reaching outside the species. Software for building and updating DB sets are described. Other comparative genomic uses for these DBs will be surveys of transposable elements and pan-genome analysis.

## Methods

### Genome assemblies for GI detection

We began with a collection of 288,451 genome assemblies downloaded from GenBank [11] in July 2019. These were placed into 13 large DBs based on NCBI taxonomy (Table 1), and the GI-mapping program TIGER v. 1.0 [1] was applied to each genome using the DB that contained it; a full report on the results will be forthcoming. We subsequently reassigned taxonomy using the system of the GTDB release 202, using the explicit GTDB species assignment when available, otherwise applying our script Speciate [2]; species assignment failed for 1831 genomes. These GTDB assignments occasionally disagreed with NCBI assignments, and showed that the large DB set had split some species, leaving 338 total genomes placed in the wrong large DB. Furthermore, 501 entire species were placed in the wrong DB. Because we were interested in the taxonomic reach of TIGER, we also excluded the genomes from the Salmonella and Campylobacter DBs, which included no reference genomes outside the genus. The above-described genomes that were unassigned, misplaced or in single-genus DBs were excluded from further analysis, leaving 195,890 properly treated genomes from 27999 of the 47895 GTDB r202 species.

### GI set

For each genome, the orthogonal GI-mapping program Islander [12] was also run, and results from TIGER and Islander were unified using the TIGER package script “Resolve”. Islander is not comparative, finding GIs that are in tRNA genes by a within-genome BLASTN-based approach. Islander may refine genome coordinates of raw TIGER calls. However, use of Islander here was effectively irrelevant for our study, because we only included GIs with TIGER support and were not evaluating genome coordinates. The properly treated genomes yielded 666,602 GI calls with any TIGER support. Because support values can be depressed for a GI in a tandem, i.e., abutting another GI at the same integration site, these were excluded from further analysis, leaving 610525 non-tandem GIs in our final GI set. The list of genomes supporting each GI (i.e., the genomes for which an uninterrupted GI integration site could be found) was collected from the TIGER output uninterrupted.txt files. Typing was performed as before [5], assigning GIs a type as either a Phage or Integrative Conjugative Element (ICE) variety or as NonPI (non-Phage, non-ICE). A new type “Reject” was introduced, applied to GI calls either with size < 5 kbp, without any serine or tyrosine integrase candidates, or with identity blocks between left and right integration sites of length > 300 bp; such calls were never found convincing in numerous spot checks and as a class can be considered putative false positives. The three most abundant types were NonPI, Phage1 and Reject (263704, 126828 and 128975 GI calls, respectively). GI data are reported in S3 File in S1 Data.

Large island-rich species were selected as having more than 300 genomes, with more than 100 each of three main island types (Phage1, NonPI, and Reject), and more than 1000 total for those three types, yielding 29 species (S1 Table in S1 Data).

### SMART DB software pipeline

The pipeline automates genome collection, DB design, and updates paralleling those of GTDB. Two modes are available: Design (blue steps in Fig 5), which designs and prepares a DB set covering all species in a GTDB release, or Quick Setup (green steps in Fig 5), where the user chooses a subset of DBs (or all) to prepare from a precalculated (downloaded) DB design file. The Design mode requires certain data from a GTDB release, and has five main conceptual steps: collecting genome assemblies, calculating pairwise distances (both between genomes within a species and between the species), ranking genomes within each species, designing and preparing each DB. The software negotiates collection of needed genome assemblies from the NCBI FTP server; since some downloads may fail in any session, collection attempts repeat until either all needed genomes are downloaded, or the number of missing genomes stops decreasing. The latter may occur when small numbers of genomes are suppressed or missing on the FTP server. Because these missed genomes may yet be available through the NCBI web site, the user is allowed to halt pipeline progress, for manual download of any desired missing genomes; however these missing genomes are not required as the software will adapt to calculate databases without them. All pairwise distances between genomes within a species are taken using Mash [13] with default settings. Pairwise distances between archaeal or bacterial species are taken from the GTDB species representative tree. The genomes of each species are ranked as follows: 1) The species representative is removed from the list, to be returned later. 2) The genome is doubly sorted for quality; first by the entry for “mimag _quality” in the GTDB metadata table (in the order high, medium, low), and then by contig count from low to high values. 3) The 10% with the lowest quality remain in place at the bottom of the list. 4) The top 90% are reordered according to diversity: the species representative is placed first in the list; the second genome is the one most distant (by Mash distance) to the first; the third genome is the one with the highest summed distance to the first and second; and so on. The SMART DB for each species, with a given cap on DB size, is designed as follows. 1) The DB is filled using the ranked list for the species. 2) If the cap is not reached, filling continues with the closest species (by tree distance), and so on with other species according to distance from the species. 3) No genomes from outside the taxonomic order are allowed, which means that some DBs do not reach the cap. After DB design is completed for each species, we observe that many species share the same DB composition as another, such that far fewer DBs need to be created than the number of species. Numerous DBs were extremely small (containing less than 5 genomes), reflecting the small genome count for certain taxonomic orders. There were 633 for the GTDB 202 release (Table 2), representing a total of 1096 species. Although these very small DBs are created without issue, they may be significantly less apt to find GIs. The program therefore warns the user that such DBs were created, and specifies them. BLASTN databases are created for each unique DB design (although this step can be omitted if only the design information is desired). This Design mode can be run *de novo* on the first GTDB release analyzed, or when updating to a new GTDB mode.

The Quick Setup mode of the pipeline does not require download of GTDB data, instead using a precalculated DB design file available at our GitHub repository (below). This mode additionally allows the user to prepare only a subset of the SMART DBs, for example when only a limited group of bacteria are under study. This abbreviated pipeline collects needed genomes and builds the DBs, skipping distance measurements and DB design, the slowest steps of the pipeline.

To assist in deciding which DB to apply to a new query genome, we recommend our utility script Speciate, which quickly determines the GTDB species of the genome. It should be noted that very small (< 200 kbp) genomes, such as *Carsonella*, are not treated by GTDB, nor by our system. Both pipeline modes prepare a Mash sketch database (for all the species whose representative genome has been collected), in support of Speciate. Software dependencies are Mash v2.3 [13] and BLAST v2.6.0 [4], with higher versions likely to be compatible; Speciate additionally requires fastANI [14] (no version assigned). The SMART DB pipeline and auxiliary scripts and data are available at github.com/sandialabs/SmartDBs.

### Benchmarking

Three genomes were selected from each large species (the top three from each ranked list) for TIGER runtime measurements for a total of 87 genomes tested. The genome annotation steps of a *de novo* TIGER run were skipped, supplying previously determined annotation files; only the BLASTN and GI merging steps were performed. For each genome, four intra-species SMART DBs were tested, capping at 200, 300, or 500 genomes, or not capping (using all genomes available for the species). A fifth DB was also tested, the original large DB. This set totaled 435 tests. From the LINUX tool “time”, the “user” and “sys” times were summed, and the cache was routinely cleared after each run to prevent cache-based artifacts.

## Supporting information

S1 Data (DOCX)

S1 Text (GZ)

S2 Text (GZ)
