# Supplementary material for: Speeding genomic island discovery through systematic design of reference database composition
Source: PLoS One. 2024 Mar 13;19(3):e0298641. doi: 10.1371/journal.pone.0298641 (PMC10936790; doi:10.1371/journal.pone.0298641)

SUPPLEMENTARY MATERIALS for “Speeding genomic island discovery through systematic design of reference database composition” by Steven L. Yu, Catherine M. Mageeney, Fatema Shormin, Noushin Ghaffari, Kelly P. Williams.

SUPPLEMENTARY FILES

**S1 File. Genome ranks.** The 197,269 genomes of the 11 large DBs are presented in four columns: 1) genome ID, 2) assembly ID, 3) species (GTDB release 202 system), 4) rank order for DB testing (rank 0 is for genomes that were excluded from DB design because they were not explicitly listed at GTDB, but were treated by TIGER). Available at

<https://figshare.com/ndownloader/files/39338453?private_link=b2bd2884033cb3122b1b>.

**S2 File. Large DB composition.** The species present in each large DB are listed. Available at

<https://figshare.com/ndownloader/files/39338450?private_link=2ec1d69e2764da8ce71a>.

**S3 File. Genomic island set.** The 610,525 GIs are presented in three columns: 1) the source genome ID, 2) the GI type, and 3) a comma-separated list of the IDs of the supporting genomes. Available at

<https://figshare.com/ndownloader/files/39337811?private_link=3b37b88d7daf6f3ea20f>.

SUPPLEMENTARY TABLES

**S1 Table. Large island-rich species studied herein**. Counts are given for genomes from the species in the large DB and the subset that are part of GTDB. Phage1, NonPI and Reject are counts for the main GI types.

| Species | Large DB | Genomes treated | Genomes assigned by GTDB | GI total | Phage1 | NonPI | Reject |
| --- | --- | --- | --- | --- | --- | --- | --- |
| *Escherichia flexneri* | EnterobacteriaceaeOther | 9932 | 9089 | 70454 | 16356 | 23037 | 23035 |
| *Escherichia coli* | EnterobacteriaceaeOther | 3474 | 3348 | 30836 | 6132 | 15440 | 6604 |
| *Escherichia dysenteriae* | EnterobacteriaceaeOther | 1603 | 1172 | 12778 | 3256 | 4116 | 4035 |
| *Escherichia coli_D* | EnterobacteriaceaeOther | 1505 | 1448 | 11082 | 2507 | 3745 | 3709 |
| *Enterobacter hormaechei_A* | EnterobacteriaceaeOther | 1037 | 1037 | 6257 | 2083 | 2259 | 1167 |
| *Klebsiella pneumoniae* | EnterobacteriaceaeOther | 7054 | 7047 | 43925 | 14072 | 15715 | 6523 |
| *Klebsiella quasipneumoniae* | EnterobacteriaceaeOther | 377 | 377 | 1990 | 516 | 883 | 288 |
| *Klebsiella variicola* | EnterobacteriaceaeOther | 331 | 331 | 1730 | 452 | 858 | 207 |
| *Cronobacter sakazakii* | EnterobacteriaceaeOther | 347 | 337 | 1311 | 468 | 605 | 133 |
| *Vibrio cholerae* | GammaproteobacteriaOther | 996 | 950 | 2706 | 177 | 2205 | 214 |
| *Vibrio parahaemolyticus* | GammaproteobacteriaOther | 980 | 979 | 3905 | 124 | 3015 | 323 |
| *Pseudomonas aeruginosa* | GammaproteobacteriaOther | 4072 | 4071 | 20309 | 3588 | 9755 | 1355 |
| *Pseudomonas_E viridiflava* | GammaproteobacteriaOther | 1321 | 1321 | 5065 | 732 | 3040 | 526 |
| *Acinetobacter baumannii* | GammaproteobacteriaOther | 3524 | 3503 | 6616 | 2347 | 2464 | 1540 |
| *Burkholderia mallei* | ProteobacteriaOther | 1545 | 1545 | 7428 | 1056 | 3284 | 1949 |
| *Streptococcus pneumoniae* | Streptococcaceae | 21056 | 8153 | 82029 | 4969 | 41695 | 20936 |
| *Streptococcus pyogenes* | Streptococcaceae | 2572 | 1907 | 5311 | 2603 | 1269 | 105 |
| *Streptococcus agalactiae* | Streptococcaceae | 1142 | 1142 | 4575 | 434 | 2345 | 421 |
| *Enterococcus faecalis* | FirmicutesOther | 1007 | 1007 | 4491 | 1354 | 1961 | 541 |
| *Enterococcus_B faecium* | FirmicutesOther | 1379 | 1376 | 5296 | 775 | 3164 | 832 |
| *Lactiplantibacillus plantarum* | FirmicutesOther | 450 | 449 | 1552 | 776 | 391 | 294 |
| *Listeria monocytogenes* | Listeriaceae | 11021 | 1605 | 14601 | 6092 | 3618 | 2866 |
| *Listeria monocytogenes_B* | Listeriaceae | 10255 | 1255 | 12271 | 6325 | 2510 | 1972 |
| *Staphylococcus aureus* | FirmicutesOther | 10337 | 10332 | 30459 | 9624 | 7399 | 8126 |
| *Staphylococcus epidermidis* | FirmicutesOther | 581 | 579 | 1298 | 308 | 517 | 229 |
| *Bacillus_A bombysepticus* | FirmicutesOther | 426 | 424 | 1695 | 407 | 557 | 484 |
| *Clostridioides difficile* | FirmicutesOther | 2012 | 1481 | 11091 | 1201 | 7361 | 453 |
| *Mycobacterium tuberculosis* | Actinobacteria | 6235 | 6226 | 38442 | 7017 | 18364 | 13024 |
| *Mycobacterium abscessus* | Actinobacteria | 1683 | 1668 | 9598 | 1741 | 5566 | 1159 |

**S2 Table. GI yield (percentage) after filtering with either of two support criteria, by GI type.** The Reject type tends to have higher losses than the Phage1, by both criteria; see for example the combined results for large species (line 30).

|  | Support Criterion Filter | Only from Outside Species | | | | Only from Within Species | | | |
| --- | --- | --- | --- | --- | --- | --- | --- | --- | --- |
|  | GI Type | Phage1 | NonPI | Reject | Phage1 | | NonPI | Reject |  |
| 1 | *Acinetobacter baumannii* | 0.13 | 1.46 | 4.16 | 28.85 | | 55.52 | 89.68 |  |
| 2 | *Bacillus_A bombysepticus* | 3.44 | 21.18 | 60.95 | 2.21 | | 6.10 | 13.22 |  |
| 3 | *Burkholderia mallei* | 0.09 | 2.01 | 7.95 | 77.37 | | 71.71 | 84.66 |  |
| 4 | *Clostridioides difficile* | 0.25 | 4.90 | 10.15 | 18.65 | | 68.75 | 45.70 |  |
| 5 | *Cronobacter sakazakii* | 0.64 | 4.46 | 6.02 | 22.01 | | 47.27 | 45.11 |  |
| 6 | *Enterobacter hormaechei_A* | 0.34 | 13.99 | 41.47 | 18.63 | | 25.14 | 10.28 |  |
| 7 | *Enterococcus_B faecium* | 0.00 | 16.43 | 24.76 | 13.55 | | 29.61 | 46.51 |  |
| 8 | *Enterococcus faecalis* | 0.00 | 1.38 | 20.52 | 66.69 | | 72.11 | 62.48 |  |
| 9 | *Escherichia coli* | 2.84 | 21.46 | 32.28 | 13.76 | | 17.44 | 37.33 |  |
| 10 | *Escherichia coli_D* | 3.07 | 47.10 | 74.87 | 1.00 | | 7.72 | 0.57 |  |
| 11 | *Escherichia dysenteriae* | 10.35 | 45.53 | 77.05 | 0.34 | | 7.24 | 5.90 |  |
| 12 | *Escherichia flexneri* | 2.45 | 11.29 | 35.68 | 2.91 | | 21.86 | 46.73 |  |
| 13 | *Klebsiella pneumoniae* | 0.50 | 4.79 | 17.74 | 1.90 | | 46.68 | 45.26 |  |
| 14 | *Klebsiella quasipneumoniae* | 11.24 | 45.53 | 46.53 | 8.53 | | 5.44 | 4.51 |  |
| 15 | *Klebsiella variicola* | 24.78 | 51.75 | 27.54 | 1.77 | | 8.51 | 23.67 |  |
| 16 | *Lactiplantibacillus plantarum* | 0.00 | 3.07 | 15.65 | 79.12 | | 90.79 | 20.07 |  |
| 17 | *Listeria monocytogenes* | 0.39 | 0.50 | 16.29 | 6.73 | | 1.33 | 18.98 |  |
| 18 | *Listeria monocytogenes_B* | 1.31 | 32.43 | 19.37 | 1.77 | | 13.90 | 27.89 |  |
| 19 | *Mycobacterium abscessus* | 0.17 | 0.63 | 1.81 | 17.75 | | 43.84 | 57.72 |  |
| 20 | *Mycobacterium tuberculosis* | 0.00 | 0.04 | 0.00 | 100.00 | | 99.13 | 53.75 |  |
| 21 | *Pseudomonas_E viridiflava* | 0.14 | 12.40 | 93.35 | 72.81 | | 60.07 | 6.27 |  |
| 22 | *Pseudomonas aeruginosa* | 0.03 | 2.85 | 4.43 | 75.39 | | 58.81 | 33.51 |  |
| 23 | *Staphylococcus aureus* | 0.07 | 0.50 | 0.85 | 18.55 | | 46.98 | 72.84 |  |
| 24 | *Staphylococcus epidermidis* | 0.32 | 19.34 | 16.59 | 76.62 | | 54.55 | 62.45 |  |
| 25 | *Streptococcus agalactiae* | 0.00 | 8.36 | 18.76 | 98.16 | | 67.89 | 70.31 |  |
| 26 | *Streptococcus pneumoniae* | 0.08 | 2.41 | 1.89 | 17.51 | | 70.38 | 65.40 |  |
| 27 | *Streptococcus pyogenes* | 0.04 | 1.18 | 12.38 | 68.57 | | 40.82 | 76.19 |  |
| 28 | *Vibrio cholerae* | 0.00 | 1.04 | 1.40 | 51.98 | | 42.13 | 88.79 |  |
| 29 | *Vibrio parahaemolyticus* | 0.00 | 1.69 | 20.12 | 25.00 | | 56.85 | 60.99 |  |
| 30 | Large Species Combined | 1.42 | 8.33 | 20.46 | 22.39 | | 50.56 | 49.04 |  |
| 31 | Full GI Set | 3.79 | 13.59 | 25.30 | 25.81 | | 46.76 | 45.05 |  |

SUPPLEMENTARY FIGURES

**S1 Fig. Survey of responses to DB size variation**. Panels A-H: The 27 large species treated as in main Figure 1. All: number of GTDB-registered genomes used for DB formation. Queries: number of genomes analyzed for island yield.


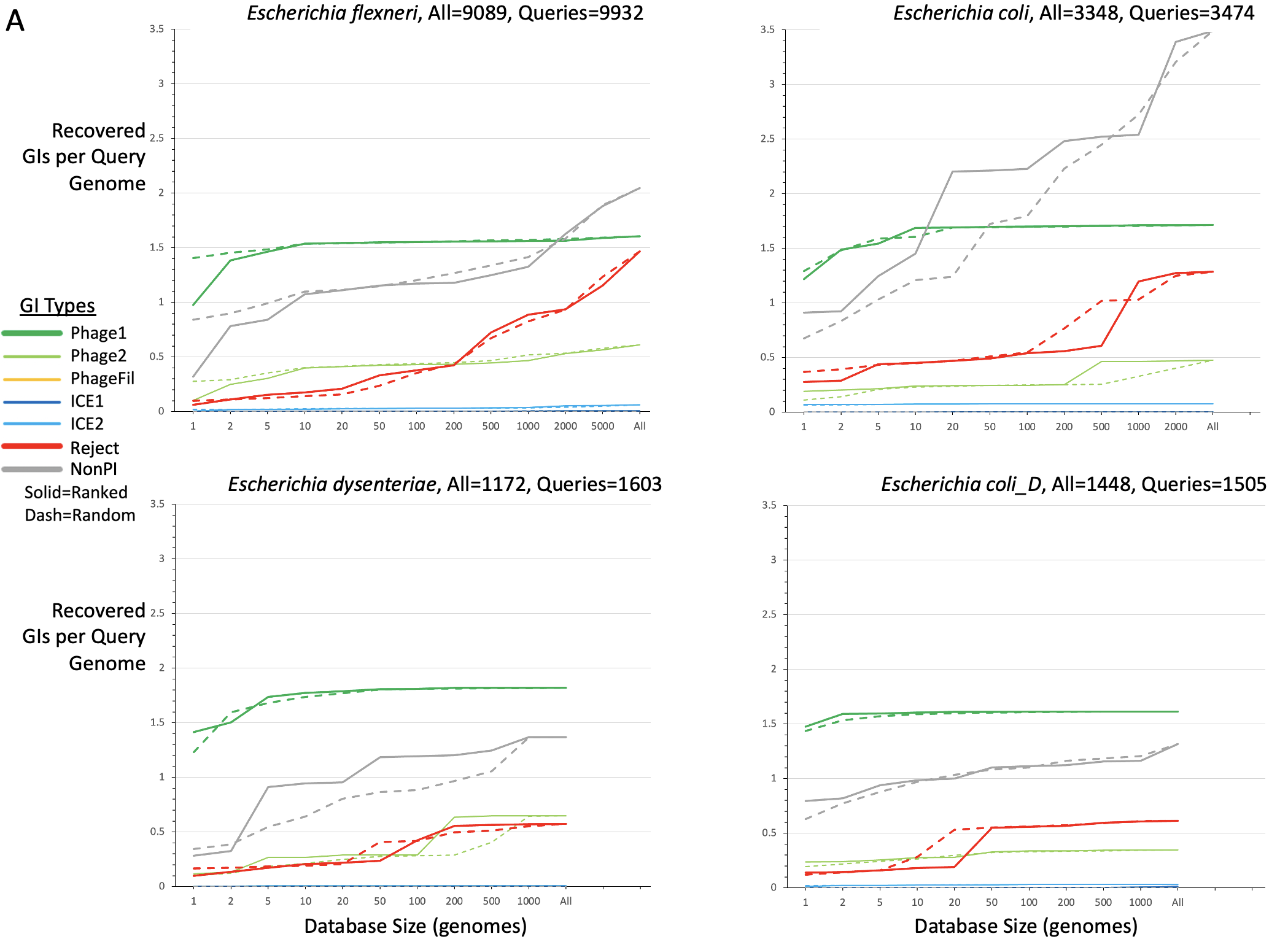


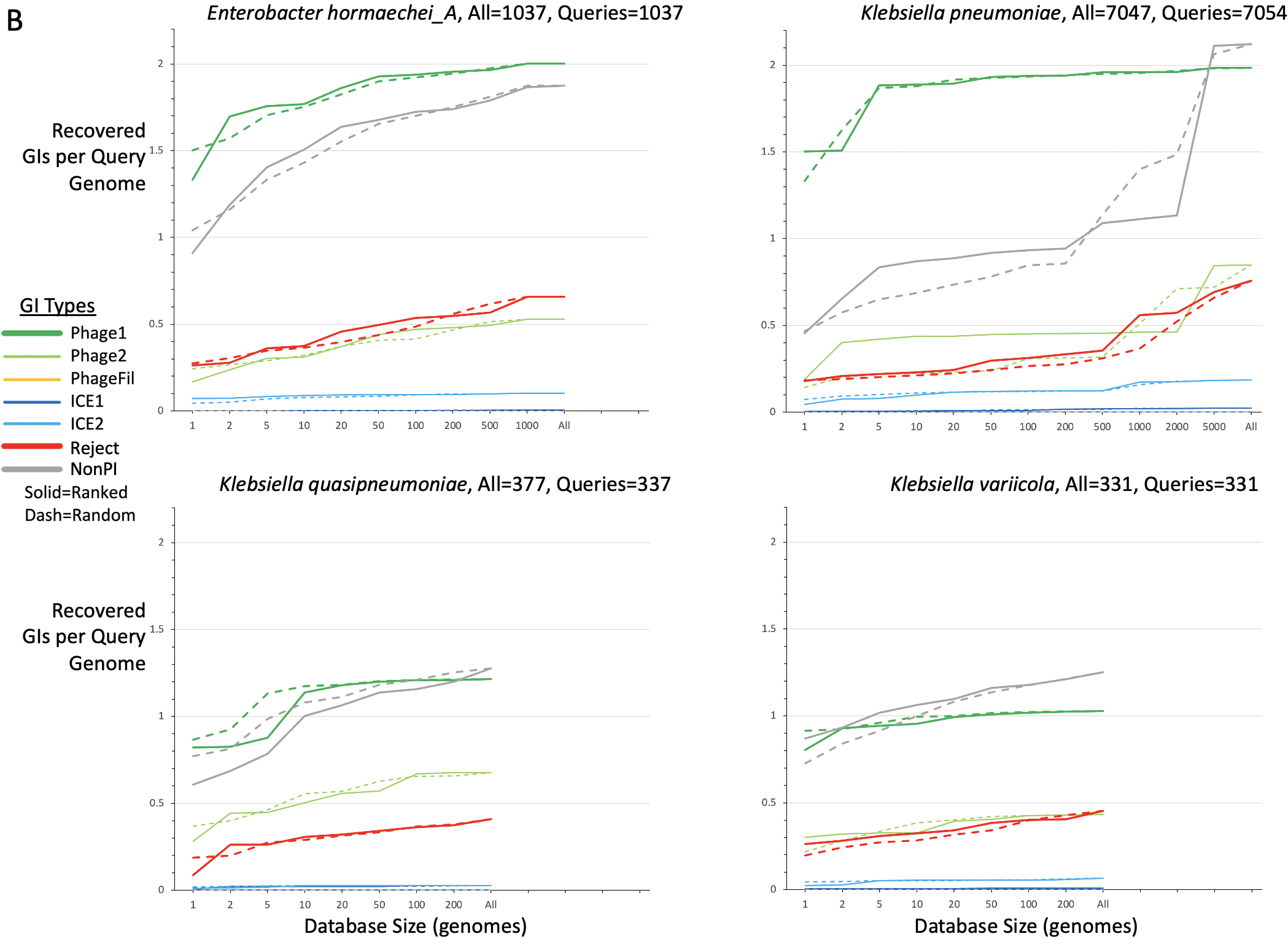


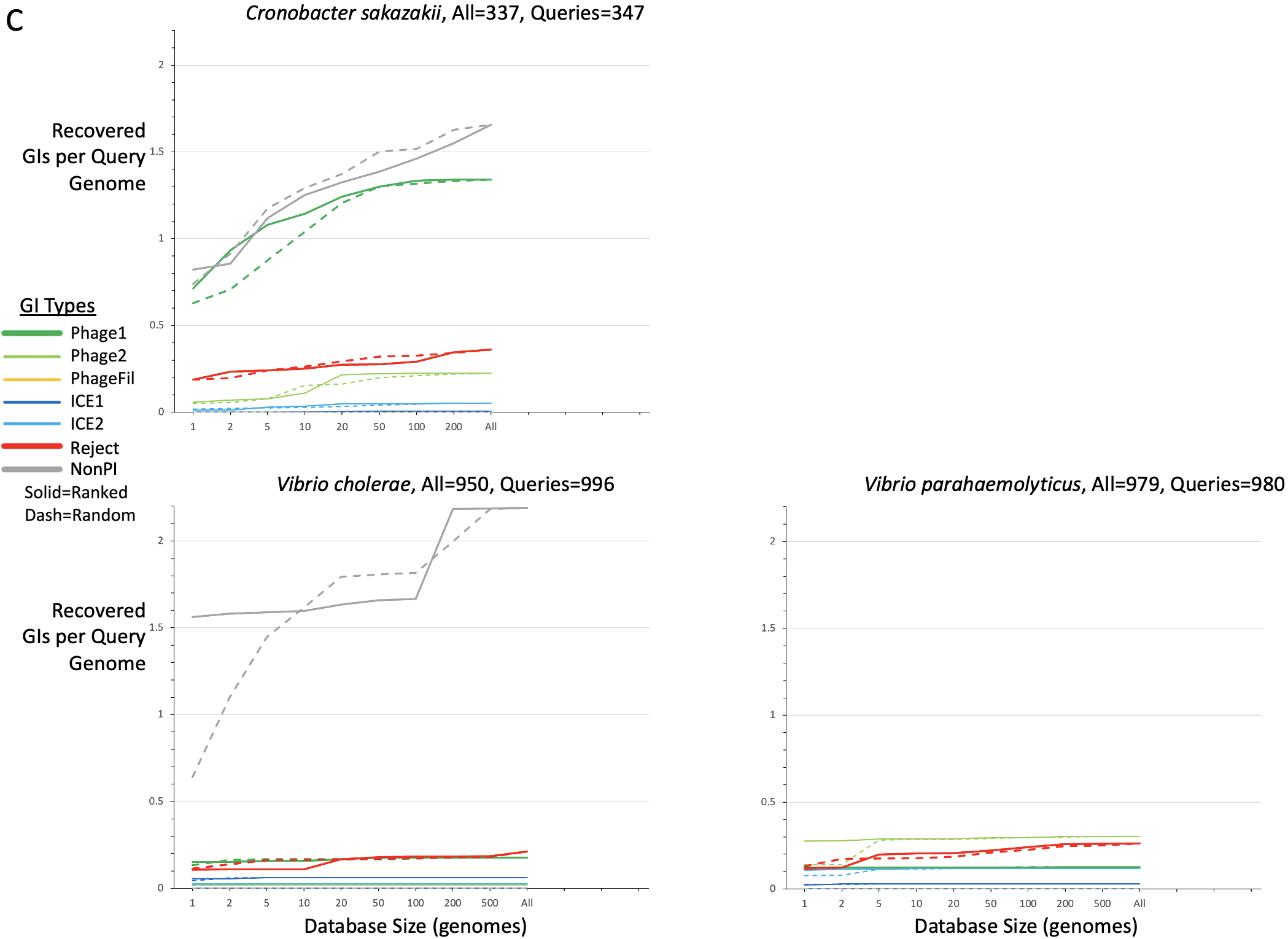


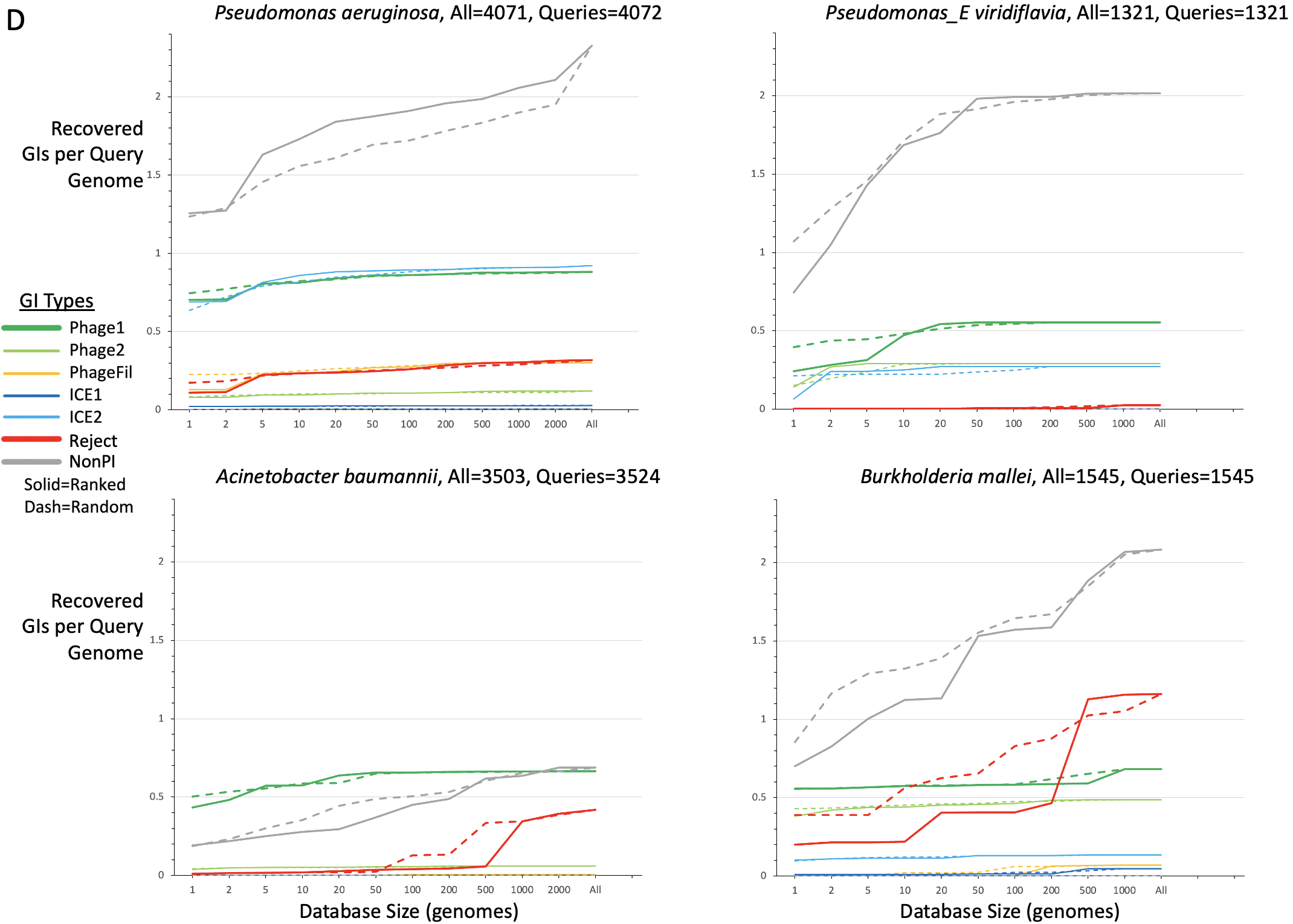


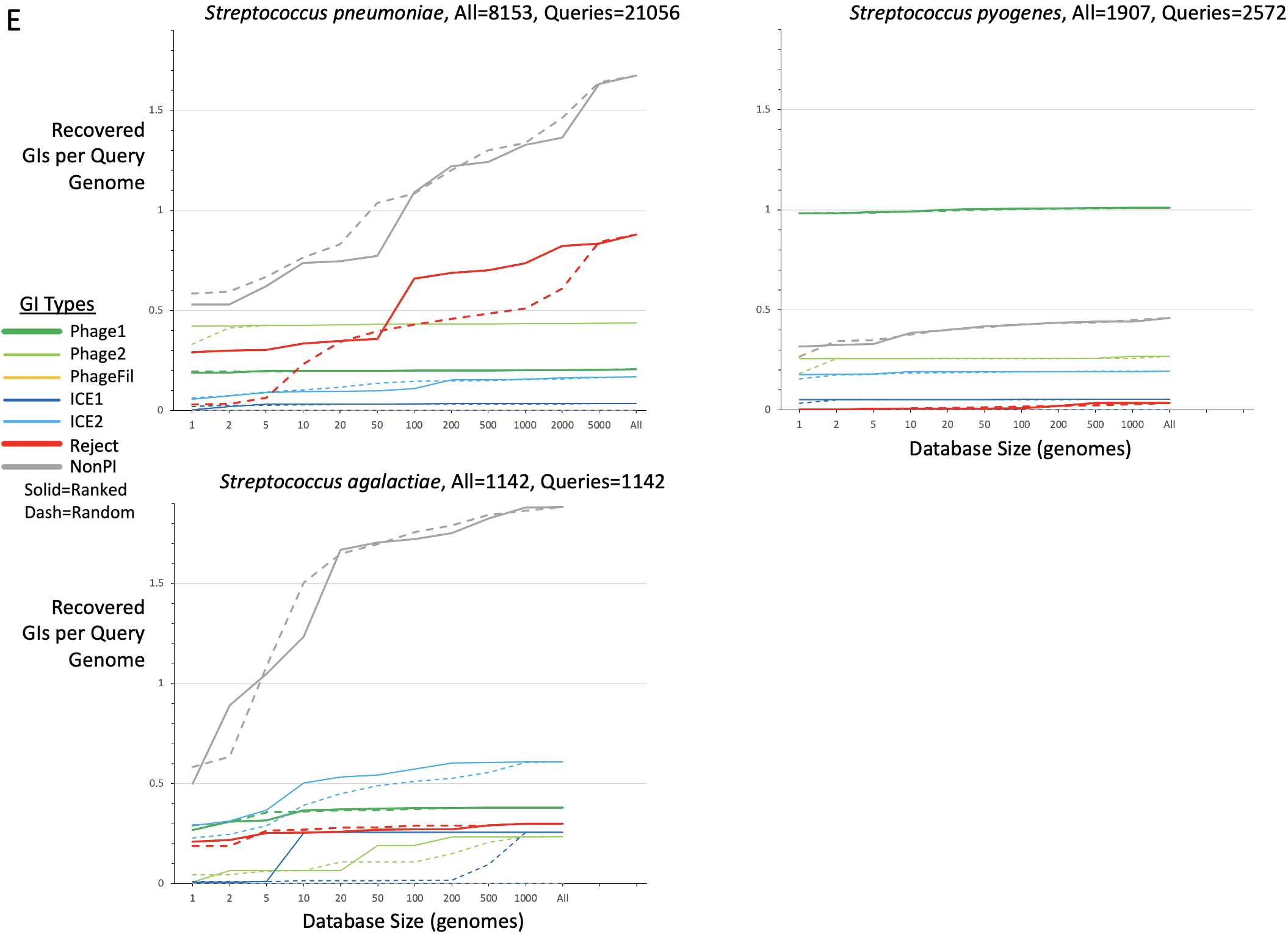


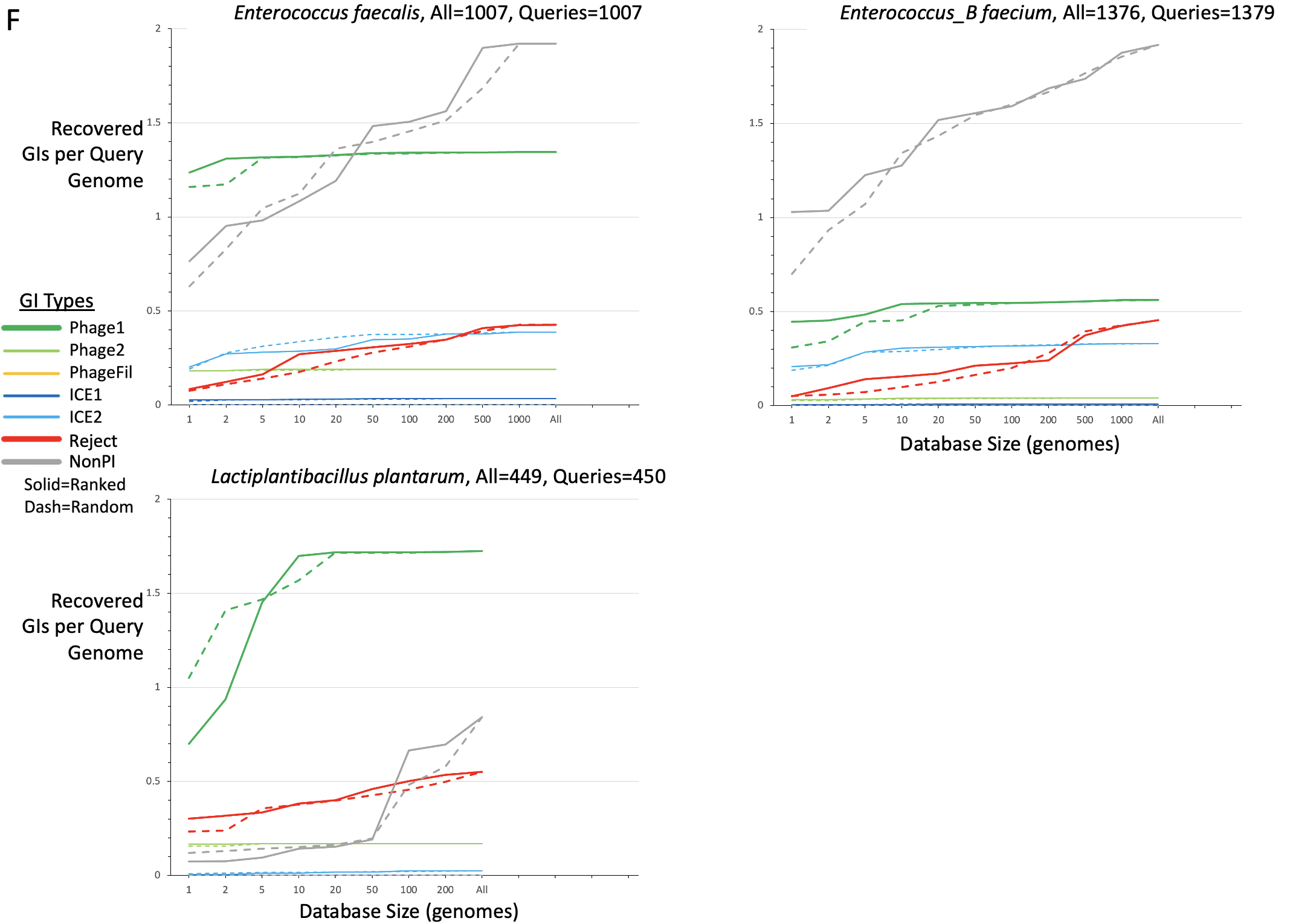


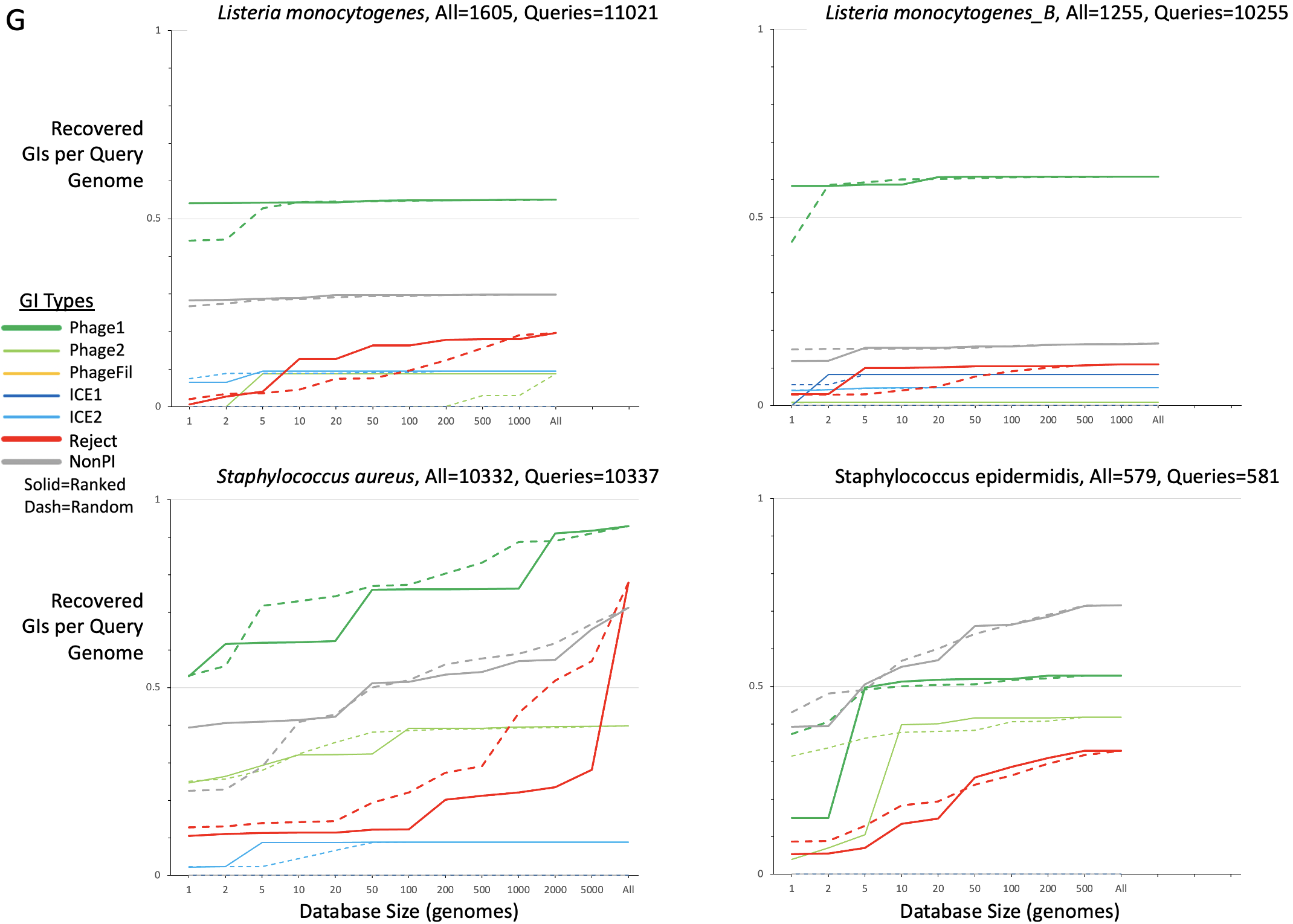


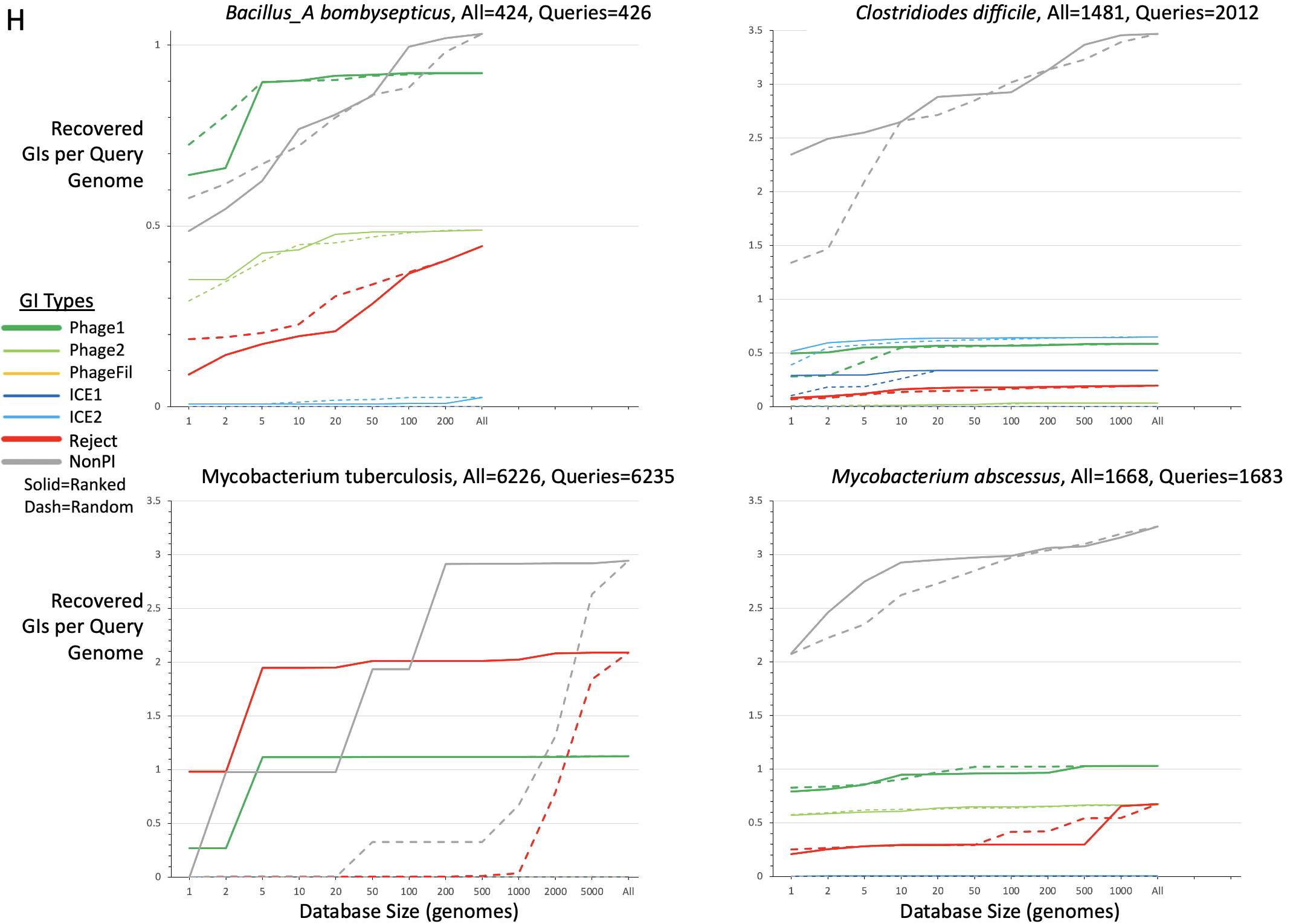


**S2 Fig. Benchmarking**. For 87 genomes (three from each large species), TIGER runtimes were measured using either (**A**) the large DB or (**B**) the 200-cap DB. Because integrase genes (*int*) are markers of GIs, TIGER launches a separate ping-pong BLASTN treatment in the vicinity of every *int* in the genome. Times were correlated with either (**B**) the *int* count for the genome, or (**A**), to roughly account for the variable size of large DBs, the *int* count multiplied by the count of genomes in the same genus as the query genome. Power law trend lines, their formulas, and R^2^ values are shown. A substantially worse R^2^ value (0.053) was obtained when *int* count alone (rather than *int* count * same-genus count) was used for the x-axis with panel **A** data. The 87 genome assembly IDs used for benchmarking were: A. baumannii (000069205, 005785665, 009759685), B. bombysepticus (006384875, 001583935, 002148155), B. mallei (000011705, 000932075, 000770565), C. difficile (001077535, 004317765, 900243255), C. sakazakii (002294395, 003955925, 000982825), E. hormaechei_A (001729745, 003056895, 002740835), E. faecalis (003319425, 902164435, 000392875), E. faecium (001544255, 000157615, 900148665), E. coli (002075295, 001614615, 003697165), E. coli_D (003009995, 000351685, 000026325), E. dysenteriae (003538515, 900195445, 002949675), E. flexneri (001256795, 000616665, 002950215), K. pneumoniae (004803565, 002261615, 000742135), K. quasipneumoniae (009647335, 000751755, 014284905), K. variicola (011394875, 000828055, 002810545), L. plantarum (009295775, 014131735, 002906095), L. monocytogenes (003607595, 900187225, 002103395), L. monocytogenes_B (001759805, 001759505, 000307025), M. abscessus (000069185, 000445035, 900136205), M. tuberculosis (004114165, 000328785, 000195955), P. aeruginosa (001457615, 000792545, 003835685), P. viridiflava (001642795, 900581105, 900588765), S. aureus (000239655, 001027105, 005146215), S. epidermidis (001476925, 006742205, 001658835), S. agalactiae (003992545, 001880675, 000186445), S. pneumoniae (001457635, 002081465, 001207355), S. pyogenes (002055535, 900982995, 900984815), V. (001402745, 001471585, 003311815), V. (003408905, 002251555, 001558495).


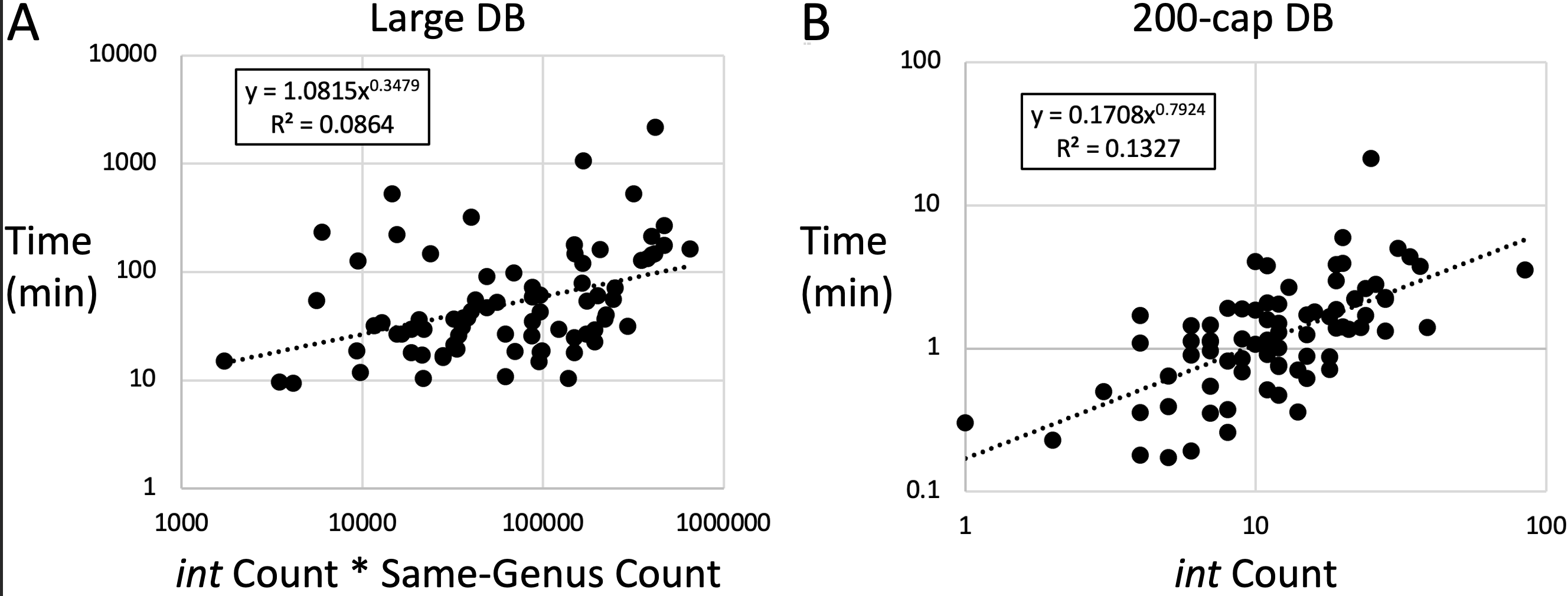

Supplement: S1 Data — (DOCX) [file pone.0298641.s001.docx]
